# LONGITUDINAL ASSESSMENT OF LOWER LIMB MUSCLE QUANTITY AND QUALITY IN ACUTE STROKE PATIENTS

**DOI:** 10.2340/jrm.v58.44630

**Published:** 2026-01-07

**Authors:** Wataru YAMAUCHI, Hiroshi AKIMA

**Affiliations:** 1 Department of Rehabilitation, Gifu Prefectural Tajimi Hospital, Tajimi, Gifu; 2 Graduate School of Medicine, Nagoya University, Nagoya, Aichi; 3 Research Center of Health, Physical Fitness and Sports, Nagoya University, Nagoya, Aichi, Japan

**Keywords:** Muscle quantity, muscle quality, ultrasonography

## Abstract

**Objective:**

This longitudinal study evaluated acute-phase stroke patients, examining changes in skeletal muscle quantity and quality. The research aimed to determine when muscle quality deteriorates, its relationship with muscle quantity, and contributing factors.

**Design:**

Prospective observational study.

**Patients:**

Forty stroke patients.

**Methods:**

Muscle quantity was assessed as the thickness of the anterior and lateral mid-thigh, while muscle quality was assessed by echo intensity of the rectus femoris and vastus lateralis. Measurements on paretic and non-paretic limbs were taken on the first day after stroke onset and on the 10th day.

**Results:**

Muscle thickness of all regions of paretic and non-paretic limbs significantly decreased at 10 days, whereas echo intensity significantly increased only in the paretic limb. A significant negative correlation between changes in muscle thickness and echo intensity was observed in the paretic limb only. Multiple regression analysis revealed that the only variable that explains the changes in echo intensity was the changes in muscle thickness of the paretic limb.

**Conclusion:**

Muscle quality begins to deteriorate as early as the acute phase of stroke. To prevent this deterioration, it is important to encourage skeletal muscle activity during the acute phase of immobilization and to minimise the reduction in muscle quantity.

## LAY ABSTRACT

This study aimed to determine the onset of skeletal muscle quality deterioration after stroke and to identify factors contributing to this change. Forty stroke patients were assessed for 10 days to evaluate change in muscle thickness, i.e., muscle quantity, and echo intensity, i.e., muscle quality, using ultrasound. Echo intensity is an index of skeletal muscle quality, with higher values indicating increased intramuscular fat and connective tissue. We found that the muscle thickness of both the paretic and non-paretic limbs decreased after 10 days, but the echo intensity increased only in the paretic limb. Importantly, patients who experienced greater decreases in muscle quantity also exhibited more pronounced deterioration in muscle quality, and the extent of change in echo intensity was closely correlated with the change in muscle thickness. These findings suggest that muscle quality deteriorates rapidly following stroke, and that preventing muscle quantity loss may be key to preserving muscle quality.

Stroke affects long-term functional impairments and limitations in activities of daily living (ADL), resulting in physical, psychological, and economic burdens for patients and caregivers (1). Although stroke mortality has declined with medical advances, favourable outcomes have shown little improvement over the past 20 years (2). Rehabilitation promotes ADL recovery, improves quality of life, and reduces economic burdens (3–5), moreover, early physical therapy (i.e., starting within 24 to 48 h after stroke) may contribute to better outcomes (6).

Skeletal muscle mass is considered to be a favourable index of mobility of stroke patients. Previous studies demonstrated that patients with reduced muscle mass during hospitalization exhibit lower walking ability even 3 months after stroke (7). Conversely, increased muscle mass is known to be associated with higher rates of discharge to home and better ADL (8, 9). Recent studies revealed that skeletal muscle undergoes not only quantitative changes but also qualitative changes after stroke, both of which may contribute to ADL recovery (10–12).

When skeletal muscle quality is evaluated from a structural perspective, it is characterized by the degree of accumulation of intramuscular adipose and/or fibrous tissue relative to the total muscle area, with higher proportions indicating poorer quality (11, 13, 14). Recent studies have reported that the deterioration of skeletal muscle quality is more strongly associated with reduced ADL levels on discharge than reductions in the quantity (15, 16). This finding suggests that muscle quality plays a key role in regaining ADL, highlighting the need to address changes in both muscle quantity and quality. Therefore, it is necessary to identify factors that may be associated with the reduction in quantity and the deterioration in quality of skeletal muscle.

According to previous studies, skeletal muscle mass was reduced in both the paretic and non-paretic limbs 7 to 9 days after stroke (7, 17–19). Additionally, reduced skeletal muscle mass is known to be influenced by factors such as stroke severity, inadequate nutritional intake, and ageing (17). In contrast, skeletal muscle quality in the paretic limb was significantly lower than that in the non-paretic limb even four weeks after stroke (10). However, the longitudinal changes in the acute phase of stroke remain poorly understood in previous studies. Previous cross-sectional research revealed that older adults with smaller muscle size show greater amounts of adipose tissue, and there was a negative correlation between muscle size and adipose tissue abundance (13). Therefore, this relationship suggests that a reduction in muscle quantity may induce deterioration in muscle quality. Moreover, skeletal muscle quality is also suspected to deteriorate during the acute phase of stroke. However, it remains unclear whether muscle quantity and quality are related longitudinally, or what factors contribute to quality changes. Understanding these changes can aid in developing measures to counteract the reduction in muscle quantity and deterioration in muscle quality. This study aimed to assess skeletal muscle quantity and quality longitudinally, to examine their relationship, and to identify associated factors.

We hypothesized that muscle quality would deteriorate in both limbs within 10 days post-stroke, associated with reduced muscle quantity.

## MATERIALS AND METHODS

**Fig. 1:**
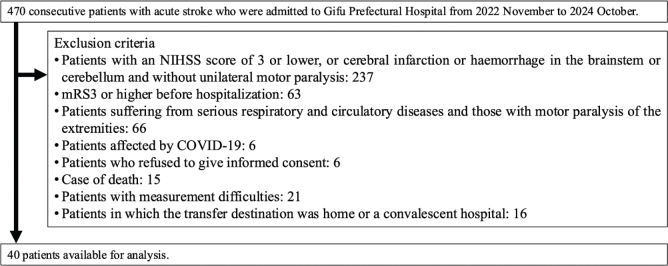
Flow diagram of patient recruitment to the study. NIHSS: National Institutes of Health Stroke Scale; mRS: modified Rankin Scale.

This prospective single-centre observational study included 40 patients who met the inclusion criteria among a total of 470 consecutive stroke patients between November 2022 and July 2024. Eligible patients were admitted within 48 h of stroke onset and had been independent in daily life (Modified Rankin Scale [mRS]: 0–2) before admission (Fig. 1). The study was approved by the Ethics Committee of Gifu Prefectural Tajimi Hospital (Approval No. 2022-19-1) and the Ethics Committee of the Health and Sports Science Center, Nagoya University (No. 22-08).

### Outcome measures

Clinical and demographic data were collected from electronic medical records and interviews with patients or families. The analysed variables included demographic factors (age, sex, body mass index, pre-admission mRS, and length of hospital stay), stroke subtypes (haemorrhagic or infarction), comorbidity (Charlson Comorbidity Index), blood biomarkers at admission (C-reactive protein, haemoglobin, and albumin), rehabilitation time per day (up to 10 days post-stroke), and nutritional status (dietary intake [20] and the pre-admission Mini Nutritional Assessment-Short Form). Additionally, physical function was assessed at the start of physical therapy using the National Institutes of Health Stroke Scale to evaluate stroke severity (21), the Brunnstrom Recovery Stage (BRS), and ultrasound-based measures of the quantity and quality of skeletal muscle. The results of these clinical and demographic characteristics are presented in Table I.

**Table I:** Characteristics of the patients

Variables	Mean	±	SD	Range
Demographic and clinical characteristics				
Age (years)	70.5	±	13.7	41.0-88.0
Sex (male/female)	25	/	15	
Stroke type (haemorrhage/infarction)	19	/	21	
Body mass index (kg/m2)	18.1	±	3.7	12.8–30.1
Pre-hospitalization MNA-SF (score)	12.7	±	2.0	
Pre-hospitalization mRS (0/1)	36	/	4	
National Institutes of Health Stroke Scale (score)	12.0	±	6.5	4.0–35.0
Charlson comorbidity index (score)	0.7	±	0.9	0.0–4.0
Motor function				
Brunnstrom recovery stage				
No voluntary movement		20		
Voluntary control achieved		20		
Rehabilitation and nutrition data				
Energy intake (kcal/kg/day)	20.9	±	8.3	0.7–37.9
Time spent on rehabilitation per day (min)	113.9	±	22.1	60.0–154.0
Length of hospital stay (day)	27.3	±	13.9	11.0–68.0
Laboratory data				
C-reactive protein (mg/dL)	0.7	±	1.6	0.0–7.3
Haemoglobin (g/dL)	13.6	±	1.9	9.3–17.2
Albumin (g/dL)	4.0	±	0.5	2.9–4.7

Values are presented as mean ± standard deviation (Range) or number of patients.

MNA-SF: Mini Nutritional Assessment-Short Form; SD: standard deviation.

### Assessment of muscle quantity and quality by ultrasound

A B-mode ultrasound device (Noblus, Hitachi Aloka Medical, Tokyo, Japan) was used to acquire images at the anterior and lateral thigh of both limbs in the supine position. Muscle thickness, as an indicator of muscle quantity, and echo intensity, as an indicator of muscle quality, were calculated from the acquired ultrasound images (22). Measurement sites were marked at 50% of the distance between the greater trochanter and the lateral knee joint fissure. The targeted muscles were the rectus femoris (RF), vastus intermedius (anterior and lateral, VI-ant and VI-lat), and vastus lateralis (VL), and they were measured twice, at the beginning of the intervention (Day 0) and 10 days later (Day 10). A linear probe (8–12 MHz) was used to measure images with the following standardized settings across patients: the frequency was set to 10 MHz, the gain and dynamic ranges were kept consistent, and the depth was adjusted to between 3 cm and 7 cm based on tissue thickness. The focus point was positioned at the top edge of all images to prevent unwanted echo intensity effects (23). To prevent rotation, knees and ankles were secured with elastic bands, maintaining 5 cm between legs. An appropriate amount of ultrasound gel was applied to minimize compression and deformation of the skin and muscles. All measurements were conducted by a single physical therapist (WY) who is well trained in ultrasound imaging. Three images were obtained from each measurement site, and the mean values of muscle thickness and echo intensity were used for analysis. Ultrasound image analysis was conducted using ImageJ software (version 1.53k; National Institutes of Health, Bethesda, MD, USA). Muscle thickness was measured in accordance with previous studies (13). The anterior thigh was defined as the RF and VI-ant, the lateral thigh as the VL and VI-lat, and the whole thigh as the average of all 4 muscles. Echo intensity was calculated for the RF and VL in the anterior and lateral thigh regions following previous studies (13). Regions of interest for echo intensity analysis excluded the fascia and were set as large as possible within the muscle belly, as described in previous research (24). The echo intensity values were calculated separately for the RF and VL, and their average was used to represent the echo intensity of the quadriceps. The inter-rater reliability was confirmed in 12 patients, yielding intra-class correlation coefficients values of 0.94–0.96 for both muscle thickness and echo intensity, with no systematic error observed.

### Statistical analysis

All variables are expressed as means (standard deviations) or the median (interquartile range). The Shapiro–Wilk test was used to assess the normality of all variables. Muscle thickness and echo intensity were analysed using a two-way repeated-measures analysis of variance (ANOVA) to evaluate the main effects of time (Day 0 vs Day 10), limb (paretic vs non-paretic), and their interaction (time × limb). When interactions or main effects were found, the Bonferroni test was used to identify significant differences. Moreover, analysis of covariance (ANCOVA) was conducted with the severity of motor paralysis (BRS < IV) (10, 25), pre-stroke mRS (0 or 1), sex (male or female), CRP (< 0.5 mg/dL) (26), and age (> 65 years) (27) as covariates. To examine the influence of motor paralysis severity, a subgroup analysis was also performed using two-way repeated-measures ANOVA after stratifying patients by BRS (No voluntary movement: Stage I–II, *n* = 20; Voluntary control achieved: Stages III–VI, *n* = 20). In addition, mean changes (Day 10–Day 0) in muscle thickness and echo intensity between the paretic and non-paretic limbs were compared using the Wilcoxon signed-rank test because the mean changes for the quadriceps on the non-paretic side did not conform to a normal distribution. The relationship between the mean changes in muscle thickness and echo intensity was assessed using Pearson’s correlation coefficient. To identify factors associated with the mean changes in echo intensity, multiple regression analysis was performed with mean changes in echo intensity for the RF, VL, and quadriceps as dependent variables, and total energy intake, National Institutes of Health Stroke Scale (NIHSS) score, mean change in muscle thickness, and age as independent variables, using the stepwise method (input criteria, *p* < 0.05; output criteria, *p* < 0.100; with the default software settings). All independent variables were assessed for multicollinearity by calculating the variance inflation factor (VIF). A significance level of *p* < 0.05 was considered statistically significant for all analyses, which were conducted using SPSS software (version 27, IBM Corp, Tokyo, Japan).

## RESULTS

### Changes in muscle thickness

**Fig. 2:**
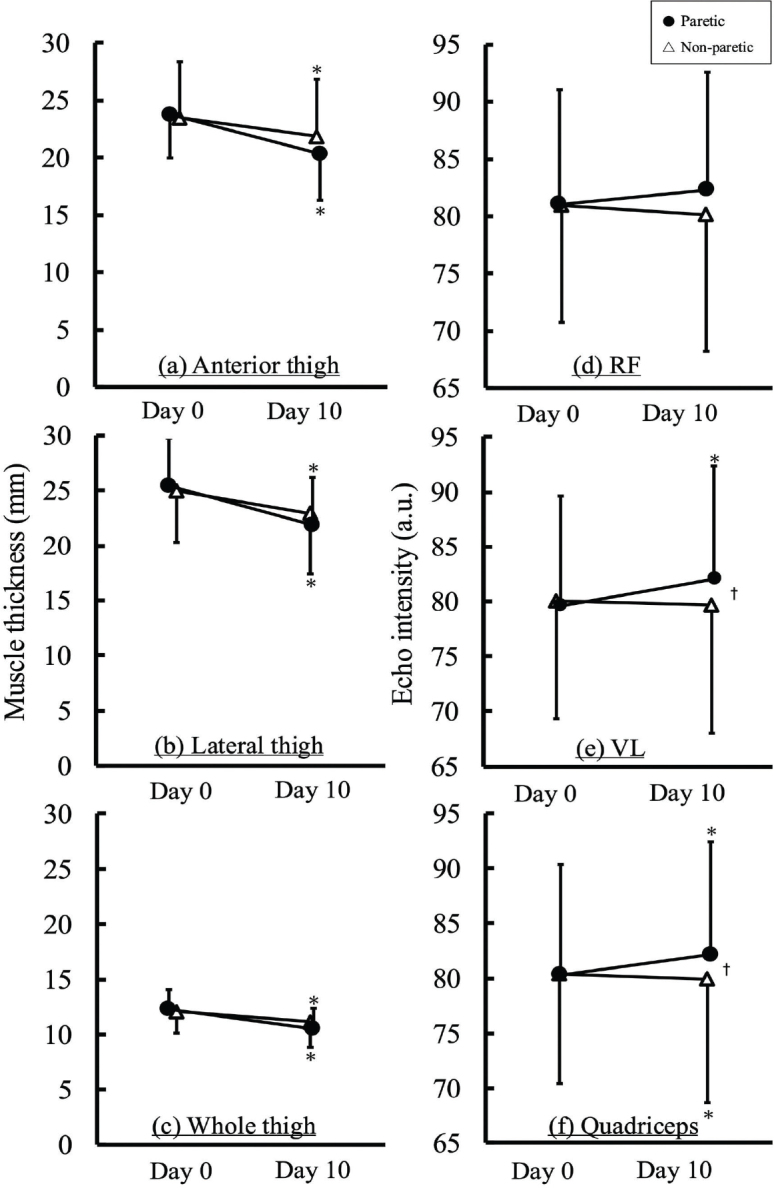
Changes in muscle thickness (a–c) and echo intensity (d–f) on Day 0 and Day 10. *p < 0.05 vs Day 0, †*p* < 0.05 vs non-paretic, a.u.: arbitrary unit.

Fig. 2 shows the changes in muscle thickness of the anterior thigh, lateral thigh, and whole thigh. In the anterior thigh, significant main effects of time (F h 37.100, *p* < 0.01, partial η² = 0.487) and limb (F = 5.635, *p* < 0.05, partial η² = 0.126) were found. Post hoc analyses revealed that muscle thickness significantly decreased from Day 0 to Day 10 (*p* < 0.01) and was significantly lower in the paretic limb than in the non-paretic limb (*p* < 0.05). For the lateral thigh, a significant time × limb interaction was found (F = 4.167, *p* < 0.05, partial η² = 0.097), with post hoc analyses revealing that muscle thickness significantly decreased from Day 0 to Day 10 in both the paretic and non-paretic limbs (*p* < 0.001, *p* < 0.002). However, no significant differences were observed between the paretic and non-paretic limbs on either Day 0 or Day 10. In the whole thigh, a significant main effect of time was found (F = 73.725, *p* < 0.01, partial η² = 0.654), with post hoc analyses revealing that muscle thickness significantly decreased from Day 0 to Day 10 (*p* < 0.01). After adjusting for covariates, a significant main effect of time was observed in the lateral thigh (F = 5.906, *p* < 0.05, partial η² = 0.148), with post hoc analyses revealing that muscle thickness decreased significantly from Day 0 to Day 10 (*p* < 0.01). Table II indicates the mean change in muscle thickness. Specifically, in the lateral thigh, the paretic limb showed a significantly greater reduction in muscle thickness than that in the non-paretic limb (*p* < 0.05). Furthermore, although reductions were also seen in the anterior and whole thigh in both limbs, these differences were not statistically significant.

**Table II:** Mean changes in muscle thickness and echo intensity between paretic and non-paretic limbs

Measurement		Paretic limb		Non-paretic limb		*p*-value
Muscle thickness (mm)	Anterior thigh	–2.85	(0.20–5.90)	–1.98	(–0.39–4.32)	0.288
	Lateral thigh	–3.22	(1.43–6.02)	–2.30	(0.01–4.28)	0.028*
	Whole thigh	–1.57	(0.74–3.16)	–1.03	(–0.15–2.19)	0.076
Echo intensity (a.u.)	Rectus femoris	2.01	(–1.25–4.67)	2.75	(–4.85–3.14)	0.062
	Vastus lateralis	3.49	(–1.78–5.68)	–1.06	(–3.82–3.10)	0.010*
	Quadriceps	2.45	(–0.73–4.74)	1.09	(–2.45–3.56)	0.010*

Values are expressed as the median (interquartile range [IQR]).

* *p* < 0.05 vs non-paretic limb (Wilcoxon signed-rank test). a.u.: arbitrary unit.

Mean changes were calculated as Day 10 minus Day 0.

### Changes in echo intensity

Fig. 2 also shows the changes in echo intensity of the RF, VL, and quadriceps. In the RF, no significant main effects of time, limb, or the time × limb interaction were found. In the VL and quadriceps, significant time × limb interactions were found (F = 7.600, *p* < 0.01, partial η² = 0.163, F = 7.824, *p* < 0.01, partial η² = 0.167). Post hoc analyses revealed that the echo intensity increased significantly in the paretic limb from Day 0 to Day 10 for both the VL and quadriceps (both *p* < 0.01). No significant change was found on the non-paretic limb in the VL, while the quadriceps showed a significant decrease (*p* < 0.01). Additionally, the paretic limb showed significantly higher echo intensities than those of the non-paretic limb on Day 10 in both the VL and quadriceps (both, *p* < 0.05), despite the lack of inter-limb differences on Day 0. After adjusting for covariates, significant time × limb interactions were found for both the VL (F = 7.365, *p* < 0.01, partial η² = 0.178) and quadriceps (F = 4.330, *p* < 0.05, partial η² = 0.113). Post hoc analyses revealed that, in both muscles, the echo intensity significantly increased on the paretic limb from Day 0 to Day 10 (VL, *p* < 0.01, quadriceps, *p* < 0.01). Although no significant differences were found between the paretic and non-paretic limbs on Day 0, the paretic limb exhibited a significantly higher echo intensity on Day 10 (*p* < 0.05, *p* < 0.01). Table II lists the mean changes in echo intensity. Specifically, the paretic limb showed a significantly greater increase in echo intensity than that of the non-paretic limb in both the VL (*p* < 0.01) and the quadriceps (*p* < 0.01). No significant difference was found in the RF.

### Echo intensity changes stratified by motor paralysis severity

In the severely paralyzed group, significant time × limb interactions were found in the RF (F = 6.685, *p* < 0.05, partial η² = 0.260), VL (F = 5.917, *p* < 0.05, partial η² = 0.237), and quadriceps (F = 10.421, *p* < 0.01, partial η² = 0.354). Post hoc analyses revealed that the echo intensity increased significantly on the paretic limb from Day 0 to Day 10 in the VL (*p* < 0.01) and quadriceps (*p* < 0.01). However, no significant changes were found on the non-paretic limb in the VL and quadriceps. Similarly, there were no significant changes in echo intensity observed in the RF in either the paretic or non-paretic limbs. Similar inter-limb differences at Day 10 were also found in the VL (*p* < 0.05) and quadriceps (*p* < 0.01), whereas no significant differences were found on Day 0 for the VL and quadriceps. For the RF, no within-limb differences were found over time in both limbs, but the paretic limb was significantly higher than the non-paretic limb on Day 10 (*p* < 0.05), despite the absence of a significant difference on Day 0.

### Correlation between echo intensity and muscle thickness

**Fig. 3:**
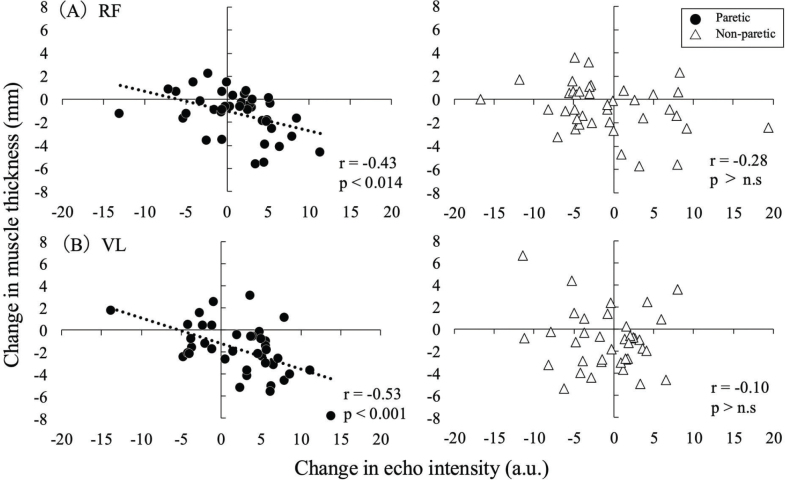
Correlations between changes in muscle thickness and echo intensity in the RF (upper) and VL (lower). A negative mean change in muscle thickness indicates a reduction in size, while a positive mean change in echo intensity reflects deterioration in muscle quality.

Fig. 3 shows the correlations between echo intensity and muscle thickness. Significant negative correlations were found between the changes in echo intensity and muscle thickness in the paretic limb for the RF (*p* < 0.01, r = –0.43) and VL (*p* < 0.01, r = –0.53). However, in the non-paretic limb, no significant correlations were found for either the RF or VL.

### Multiple regression analysis

Table III gives the results of the multiple regression analyses. All independent variables were assessed for multicollinearity by calculating the VIF, and no variable exceeded the commonly accepted threshold of 5. Multiple regression analysis revealed that the RF, VL, and quadriceps in the non-paretic limb, and the VL in the paretic limb, were not selected as explanatory variables and were excluded from the model. In contrast, in the paretic limb, changes in muscle thickness were significantly associated with changes in echo intensity for the RF and quadriceps (RF, R^2^ = 0.184, adjusted R^2^ = 0.163, *p* < 0.006, quadriceps, R^2^ = 0.099, adjusted R^2^ = 0.075, *p* < 0.05)

**Table III:** Results of multiple regression analyses

Table	Dependent variable	Independent variables	β	SE	R	R^2^	Adjusted R^2^	*p* value
RF_EI	Paretic	Mean change in muscle thickness	-1.034	0.353	0.429	0.184	0.163	*0.006
	Non-paretic	Excluded						
VL_EI	Paretic	Excluded						
	Non-paretic	Excluded						
Quadriceps_EI	Paretic	Mean change in muscle thickness	-0.496	0.243	0.314	0.099	0.075	*0.049
	Non-paretic	Excluded						

EI: echo intensity, SE: standard error.

* *p*<0.05.

## DISCUSSION

This longitudinal study investigated the changes in skeletal muscle quantity and quality in stroke patients during a 10-day hospitalization. The main findings of this study were that a significant increase in echo intensity was observed only for the paretic limb 10 days post-stroke, and the mean changes in echo intensity in the paretic limb were significantly associated with changes in muscle thickness. These findings suggest that the deterioration in skeletal muscle quality in the paretic limb is consistent with our hypothesis, highlighting the importance of preventing muscle quantity loss to mitigate a further deterioration in muscle quality.

Echo intensity is an index of ultrasound images intensity, and it has been widely used in various studies (13, 22). Echo intensity was shown to correlate moderately with intramuscular fat values obtained by magnetic resonance imaging (MRI) and computed tomography (CT) (22, 28). Previous studies showed that echo intensity was significantly higher in the paretic than in the non-paretic limb 4 weeks after stroke (10), suggesting that deterioration in skeletal muscle quality took place several weeks post-stroke. However, how skeletal muscle quality changes in relation to muscle quantity in the acute phase of stroke (Day 0–Day 10) has remained unclear. This study demonstrated that echo intensity significantly increased in the VL and quadriceps in the paretic limb after only 10 days since beginning the measurement (see Fig. 2), with the effect remaining significant in the VL and quadriceps even after adjusting for covariates. Furthermore, when classified by motor paralysis severity, increased echo intensity was also observed only in the severely paralyzed group (BRSI II), whereas no significant change in echo intensity was found in the mild to moderately paralyzed group (BRS III–VI). These results suggest that the increases in echo intensity occurred exclusively in the paretic limb and only in patients with severe motor paralysis. The paretic lower limb, affected by motor paralysis, receives markedly reduced neural input because of central motor pathway disruption (29). Consequently, a significant decline in voluntary muscle activity occurs, which in turn leads to a greater reduction in skeletal muscle mass than that in the non-paretic limb (see Table II). Because the echo intensity is an index associated with extracellular lipids measured by H-magnetic resonance spectroscopy, it therefore particularly reflects adipose tissue accumulated in the muscle cells’ extracellular space (22). On the basis of these considerations, the results of this study suggest that it is possible that the rapid reduction in skeletal muscle mass in the paretic limb led to the infiltration of adipose tissue into the extracellular space, which in turn manifested as an increase in echo intensity. In this study, a significant negative correlation was observed between the changes in muscle thickness and echo intensity in the paretic limb (see Fig. 3), supporting this interpretation. According to previous studies, the possibility of short-term physical inactivity may induce adipose and connective tissue accumulation within the muscle. Pagano et al. (2018) demonstrated that the expression of mature adipocyte markers such as perilipin and FABP4 increased after a short period of bed rest, suggesting that the development of adipose tissue in a certain muscle can begin after only a few days of inactivity (30). This finding may support the early increase in echo intensity, if changes in adipose tissue and echo intensity occurred on a similar time scale in the paretic limb to that observed in the present study. This result indicates that muscle quality deterioration may progress rapidly even in the acute phase of stroke. In contrast, no change in the echo intensity in the non-paretic limb was observed during the observation period, which indicates that muscle quality was maintained. This finding was inconsistent with our hypothesis that an increases echo intensity in the non-paretic limb would also be observed. This discrepancy could be explained by the compensatory overuse of the non-paretic limb in daily activities. In this study, skeletal muscle mass was significantly reduced in both the paretic and non-paretic limbs, but the extent of the reduction was relatively small on the non-paretic limb (see Table II). In stroke patients, the frequency of paretic limb use decreases, whereas the non-paretic limb is frequently used because of compensatory overuse (31). Therefore, increased use of the non-paretic limb may have led to greater muscle activity, which potentially contributed to mitigating the reduction in muscle quantity and the deterioration in muscle quality (32).

According to previous cross-sectional studies, changes in the quantity and quality of skeletal muscle showed an inverse relationship (13, 14). For instance, Akima et al. demonstrated a significant negative correlation between muscle thickness and echo intensity in healthy older adults (13, 14). Similarly, Maeda et al. identified muscle thickness as a significant independent variable explaining the variation in echo intensity in the paretic limb in stroke patients during the recovery phase (11). These studies indicate that there is a close relationship between the quantity and quality of skeletal muscle. However, it is unclear whether there are significant associations between skeletal muscle quantity and quality with longer observation periods for patients. We found that there were significant negative correlations between the changes in muscle thickness and echo intensity in the RF and VL in the paretic limb (see Fig. 3). This finding indicates that a greater reduction in muscle thickness was associated with a greater increase in echo intensity, suggesting that the decline in muscle quantity and the deterioration in muscle quality may progress in tandem. These results support previous findings (13, 14) and demonstrate that the reduction in the quantity and deterioration in the quality of skeletal muscle are inversely related, even in a longitudinal study.

We performed multiple regression analyses to identify factors associated with the mean change in echo intensity and found that a decrease in skeletal muscle quantity was a significant explanatory variable. These findings indicate that preserving muscle thickness is important for preventing changes in echo intensity. Previous studies reported that increases in echo intensity were more strongly associated with a decline in physical function than reductions in muscle thickness in patients admitted to intensive care units (33). Similarly, in patients with subacute stroke, echo intensity has been shown to be more closely related to functional recovery, such as gait ability, than muscle thickness (15, 16). These findings suggest that qualitative changes in muscle might affect independence in ADL more negatively than quantitative changes do. Therefore, it is important to prevent reductions in muscle thickness and to suppress increases in echo intensity from the acute phase of stroke. In physical therapy, early-phase interventions aimed at maintaining muscle mass may be effective strategies to prevent increases in echo intensity. Such interventions include appropriate nutritional support (34), neuromuscular electrical stimulation (19), and the promotion of physical activity (35).

There are three limitations in this study to address. First, echo intensity was used as an index of muscle quality, as it is known to reflect intramuscular fat and/or connective tissue (22, 28). However, echo intensity can be influenced by various factors, such as the thickness of adipose tissue and muscle itself, water content distribution, and age (36, 37). Therefore, we need to use caution in interpreting the changes in echo intensity. Future studies should use advanced imaging techniques such as CT or MRI for a more detailed evaluation of intramuscular fat and connective tissue. Second, 40 patients were enrolled from one hospital, which might limit the generalizability of the findings. Larger, multi-centre studies are needed to validate the results. Finally, we did not provide any reports regarding the physical activity levels of the patients in this study. Almost all stroke patients are known to be physically inactive, which has been reported in previous studies (18, 38). However, because of the lack of data on physical activity levels, it was difficult to evaluate the impact of physical activity on the changes in the quantity and quality of skeletal muscle. Nonetheless, significant correlations between physical activity levels and both muscle thickness and echo intensity have been observed in stroke patients (35). This limitation might be of help for our future studies, which should incorporate objective physical activity measurements to better understand the influence on changes in the skeletal muscle quantity and quality.

In conclusion, we assessed the longitudinal changes in skeletal muscle quantity and quality during the acute phase of stroke. This study aimed to determine when muscle quality begins to deteriorate, how it relates to muscle quantity, and which factors may contribute to its deterioration. The echo intensity of the paretic limb increased significantly within 10 days after stroke onset. Importantly, a significant negative correlation was observed between the changes in muscle thickness and echo intensity in the paretic limb, and the changes in muscle thickness were also identified as a significant explanatory variable for the echo intensity changes. These findings suggest that skeletal muscle quality deteriorates in the acute phase of stroke, highlighting the importance of preventing muscle quantity loss to mitigate the deterioration in muscle quality. Promoting muscle activity and/or preventing a decrease in muscle size from the acute phase could contribute to the preservation of muscle quality of patients with stroke.
